# The Protective Effects of *Areca catechu* Extract on Cognition and Social Interaction Deficits in a Cuprizone-Induced Demyelination Model

**DOI:** 10.1155/2015/426092

**Published:** 2015-02-26

**Authors:** Abulimiti Adilijiang, Teng Guan, Jue He, Kelly Hartle, Wenqiang Wang, XinMin Li

**Affiliations:** ^1^Xia Men Xian Yue Hospital, Xian Yue Road 387-399, Xiamen 361012, China; ^2^Department of Psychiatry, Faculty of Medicine and Dentistry, University of Alberta, 1E7.31 Walter C. Mackenzie Health Sciences Centre, Edmonton, AB, Canada T6G 2B7; ^3^Department of Human Anatomy and Cell Science, Faculty of Medicine, University of Manitoba, 745 Bannatyne Avenue, Winnipeg, MB, Canada R3E 0J9

## Abstract

Schizophrenia is a serious psychiatric illness with an unclear cause. One theory is that demyelination of white matter is one of the main pathological factors involved in the development of schizophrenia. The current study evaluated the protective effects of *Areca catechu* nut extract (ANE) on a cuprizone-induced demyelination mouse model. Two doses of ANE (1% and 2%) were administered orally in the diet for 8 weeks. Animals subjected to demyelination showed impaired spatial memory and less social activity. In addition, mice subjected to demyelination displayed significant myelin damage in cortex and demonstrated a higher expression of NG2 and PDGFR*α* and AMPK activation. ANE treatment not only significantly enhanced cognitive ability and social activity, but also protected myelin against cuprizone toxicity by promoting oligodendrocyte precursor cell (OPC) differentiation. In addition, ANE treatment demonstrated significant dephosphorylation of AMPK*α*, indicating a regulatory role for ANE in schizophrenia. This study showed that ANE treatment may enhance cognitive ability and social activity by facilitating OPC differentiation and protecting against myelin damage in cortex. Results also suggest the AMPK signaling pathway may be involved in this process.

## 1. Introduction

Schizophrenia is a serious psychiatric illness that affects about 1% of the population [1]. The symptoms of schizophrenia include “positive symptoms” (hallucinations, delusions, and disorganized thinking), “negative symptoms” (diminished emotional responses, social withdrawal, blunted affect, scarcity of speech, and lethargy), and cognitive symptoms (attention deficits, impaired executive function, and memory). Schizophrenia is responsible for enormous healthcare and nonhealthcare related expenditures worldwide. Although antipsychotic drugs acting through multiple receptors have been revolutionary for controlling positive symptoms, negative symptoms and cognitive deficits remain relatively unaffected. The development of novel antipsychotic drugs has been limited due to the relatively poor understanding of the neuropathology of this disorder [2]. Recently, increasing evidence suggests that oligodendrocytes (OL) play an important role in the pathogenesis of schizophrenia [3]. Therefore, neuroprotective agents that directly target white matter injury might be a therapeutic target for schizophrenia.

In the search for new therapeutic agents to treat neurological disorders, there has been significant progress in the area of medicinal plants. Areca nuts (from* Areca catechu*, L.), popularly known as “betel nuts,” are widely used in traditional Chinese medicine for treatment of constipation, oedema, beriberi, and dyspepsia. Areca nuts produce a variety of pharmacological effects such as heightened alertness and euphoria, [4]. Scientific studies conducted on the Areca nut have shown that it possesses antioxidant [5], antischizophrenic [6–10], memory-protective [11], analgesic, and anti-inflammatory [5] effects. Phytochemical analysis of the crude extract and the aqueous fraction demonstrated the presence of alkaloids (i.e., arecaidine, arecoline, guvacine, and guvacoline), anthraquinones, coumarins, flavonoids, saponins, sterols, hydroxychavicol, procyanidins, tannins, terpenes, and gallic acid [12]. Several studies have shown that schizophrenic patients who chewed betel nuts scored significantly lower on both positive and negative symptom scales than nonchewers on the Positive and Negative Syndrome Scale (PANSS) [6–10]. The hypothesis is that* Areca catechu* nuts may exert their beneficial effects on schizophrenia through the main active ingredient, arecoline. However, the antischizophrenic effect of* Areca catechu* nuts and their mechanism remains unclear.

Based on pharmacological properties of* Areca catechu* nuts, it was proposed that* Areca catechu* nut extract (ANE) might ameliorate schizophrenic symptoms by targeting of oligodendrocytes (OLs) to prevent demyelination of white matter. To test this hypothesis, we used a cuprizone- (CPZ-) induced demyelination animal model. With the goal of a therapeutic intervention for schizophrenia, the current study was designed to evaluate the effects of ANE on cognition, social interaction, OL restoration, and myelin repair in CPZ-induced demyelination.

## 2. Materials and Methods

### 2.1. *Areca catechu* Nut Extraction

The* Areca catechu* nut extract was provided by the TCM department of the Xianyue Hospital in Xiamen (China). Fresh* Areca catechu* nuts (2 kg) were purchased from Xiamen Chinese Traditional Medicine Hospital and authenticated by a pharmacist (Feng Tang) in TCM Department of Xiamen Xianyue Hospital, where a voucher specimen (20120910) was deposited. Briefly,* Areca catechu* nuts were cleaned of adulterants, crushed to open up the crest of the seed and soaked in 4 L of 50% aqueous-ethanol for cold maceration for a period of 7 days at room temperature. The solution was filtered through Whatman qualitative filter paper (Grade 1) and the filtrate was collected. The crushed nuts were resoaked and refiltered twice. The combined filtrates were concentrated in a rotary evaporator at 40°C under reduced pressure to yield a viscous, dark brown* Areca catechu* crude extract (Ac.Cr) weighing 200 g (yield 10% w/w). This extract was dried in an oven at 90°C and then stored at −4°C until use. It was mixed into powered mouse chow at concentrations of 1% and 2% on the day of the experiment.

### 2.2. Animals and Experimental Manipulations

Male C57BL/6 mice (7 weeks old) were purchased from Charles River Canada (Montreal, Canada). After 1 week of acclimation, the mice were fed a standard rodent chow (LabDiet, PMI Nutrition International, LLC, Brentwood, MO, USA). The milled chow contained 0.2% CPZ (w/w) (Sigma-Aldrich, St. Louis, MO, USA) for eight weeks to induce demyelination [13]. The control mice received standard rodent chow without CPZ. The animal studies in this study were performed within the guidelines set by the Canadian Council on Animal Care (CCAC). The experimental protocol was approved by the University Committee on Animal Care and Supply (UCACS) and the University of Manitoba Animal Care Committee.

C57BL/6 mice were divided into the following four groups (*n* = 10/group): control (regular chow and distilled water for 9 weeks); CPZ (distilled water and regular chow for one week and 0.2% CPZ chow for 8 weeks); and CPZ + ANE1 and CPZ + ANE2 (distilled water and regular chow for first week and 0.2% CPZ + ANE (1% or 2%) chow for 8 weeks). Each group was separately caged.

### 2.3. Behavioral Testing

#### 2.3.1. Spatial Working Memory Test

Spatial working memory was evaluated by measuring spontaneous alternation behavior in a *Y*-maze. This test is based on the natural tendency of rodents to explore novel environments and to recall which areas have been explored. The *Y*-maze has three arms (each 30 cm long), marked A, B, and C. Mice were placed individually at the end of one maze arm and allowed to roam freely for 8 min. The total number of arm entries and the series in which they occurred were recorded. Overlapping entrance sequences (e.g., ABC and BCA) were defined as the number of alternations. Alternation was calculated as a percentage:

(1) Percent  alternation =number  of  alternationstotal  number  of  arm  entries−2×100;

see [21].

#### 2.3.2. Sociability and Social Preference Tests

The sociability test was used to examine whether the test mouse preferred social interaction with unfamiliar conspecific animals to a test area without animals. This test was adapted from the three-chamber paradigm test known as Crawley's sociability [14] with several previously reported modifications [15]. The test was performed in a novel environment unknown to the test mouse. This novel environment was a cage with three interconnected compartments. The dimensions of each compartment were 23.3-cm long, 40-cm wide, and 22-cm high. The walls dividing the compartments had rectangular openings (10-cm wide and 10-cm high) allowing movement between the compartments.

To initiate the sociability test, the test mouse was placed in the central compartment (empty). In the left compartment, a conspecific stranger mouse (CSM) to the test mouse was placed under a small wire cage (diameter 11.0 cm). CSM were randomly selected from mice of the same age maintained in separate cages. In the empty right compartment (empty cage, EC), an empty wire cage of the same size and shape was placed. After a 5 min habituation period, the 10 min sociability test was performed. Sociability index (SI) was defined as the ratio of either frequency or duration between CSM side and EC side, as previously described [15].

Following the sociability test, a social preference test was performed in the same cage. In this test, the CSM in the left compartment contained a familiar mouse (FM) and a stranger mouse (novel mouse, NM) was placed in a wire cage in the right compartment. As before, the test mouse was placed in the empty central compartment, and the 10 min social preference test was conducted. The social preference test evaluates the duration of social interaction (i.e., time spent) with either FMNM mice by the test mouse. Social preference index (SPI) was defined as the ratio of either frequency or duration between novel side and familiar side, as previously described [15]. The duration and direction of mouse movements were recorded and analyzed. The NM was also randomly selected from separate group of mice of the same age. The NM had not been used in any previous experiments.

### 2.4. Tissue Preparation and Immunohistochemical Staining

At the end of the behavior tests, mice were anesthetized with sodium pentobarbital (50 mg/kg) and perfused intracardially with phosphate-buffered saline (PBS) followed by 4% paraformaldehyde in PBS. The brains were postfixed overnight in 4% paraformaldehyde. The fixed brains were rinsed three times with 0.01 M PBS, chilled in 30% sucrose at 4°C for one day, and frozen at −80°C. Serial coronal sections (30 *µ*m) of brains were cut using a sliding microtome (Thermo; Kalamazoo, MI, USA).

Frozen sections (30 *μ*m) were incubated with 0.3% H_2_O_2_ in 0.01 M PBS for 30 min at room temperature (RT) to quench endogenous peroxidase activity. The sections were then blocked with 10% goat serum in PBS or 10% rabbit serum (for MBP staining) for 1 h at RT. After blocking, the sections were incubated at 4°C overnight with the primary antibodies in the blocking solution. After rinsing, the sections were incubated for 1 h at RT with the selective biotin-conjugated secondary antibody (1 : 1000; Vector Laboratories, Burlingame, CA, USA). Staining was performed with an avidin-biotin complex kit (Vector Laboratories, Burlingame, CA, USA) and visualized with using 0.025% 3,3-diaminobenzidine as the chromogen (DAB, Sigma-Aldrich, St. Louis, MO, USA).

Goat polyclonal antibodies directed against myelin basic protein (MBP) (1 : 500; Santa Cruz Biotechnology, CA, USA) were used to detect myelin components. Rabbit polyclonal NG2 antibodies (1 : 250; Chemicon, Temecula, CA, USA) and PDGFR*α* antibodies (1 : 250; Santa Cruz Biotechnology, CA) were used as a marker for oligodendrocyte progenitor cells (OPCs) [16, 17].

### 2.5. Western Blot Analysis

The frontal cortex samples were homogenized in radioimmunoprecipitation assay lysis buffer (50 mM Tris, 150 mM NaCl, 1% NP40, 0.5% sodium deoxycholate, and 0.1% sodium dodecyl sulfate with freshly added protease inhibitor cocktail (Sigma, St. Louis, MO, USA). The supernatant was collected after centrifugation at 12,000 rpm for 10 min at 4°C. A BCA protein kit (Pierce, Nepean, Ontario, Canada) was used to quantify total protein concentration. Samples of the proteins were loaded onto 10% sodium dodecyl sulfate-polyacrylamide gel and subjected to electrophoresis at 70 V and 110 V. The proteins were then transferred to polyvinylidene fluoride membranes. The membranes were incubated in a blocking solution (5% skim milk in PBS) for 1 h at 22°C, followed by incubation with the primary antibody in a blocking solution.

Anti-MBP (1 : 5000) (Santa Cruz Biotechnology, CA, USA) was used to detect MBP. Anti-PDGFR*α* (1 : 2500) (Santa Cruz Biotechnology, CA, USA) was used to detect PDGFR*α* as marker of OPCs.

The membranes were then washed with PBS three times, followed by incubation in the blocking solution containing the rabbit anti-goat, goat anti-mouse, or goat anti-rabbit secondary antibodies (1 : 10,000). After three rinses in PBS, the immunoreactive bands were developed using an ECL detection kit (Amersham Biosciences, Baie d'Urfe, Quebec). To confirm the equal amounts of loading samples, *β*-actin was also labeled (1 : 5000; Sigma, St. Louis, MO, USA) through the same procedures as described above. Phospho-5′ AMP-activated protein kinase (AMPK*α*) (Thr172) antibodies (1 : 1000) and AMPK*α* antibodies (1 : 1000) (Cell Signaling Technology, Inc., Danvers, MA, USA) were incubated with frontal cortex samples in 5% w/v BSA, 1X Tris-buffered saline, and 0.1% Tween-20 at 4°C with gentle shaking, overnight. After incubation in a secondary anti-rabbit antibody (1 : 5000) diluted in 5% w/v BSA, 1X Tris-buffered saline, and 0.1% Tween-20, the immunoreactive band was visualized using an ECL detection kit (Amersham Biosciences, Buckinghamshire, UK).

### 2.6. Image Analysis

To quantify the number of cells expressing NG2 and PDGFR*α*, three digital images of coronal sections from each animal (including the midline and the two edges of the corpus callosum (CC) in each section) were examined. The positive cell counts are expressed as the average of positive cells in two areas of three coronal sections, 500 *μ*m apart, between 1 and −1 mm from the bregma. The averages were calculated from at least eight mice per group.

Three digital images from coronal sections (including cerebral cortex) of each animal were analyzed for MBP staining. A minimum of eight animals were analyzed in each group. The percent area of MBP-positive staining was calculated in a selected area of the cortex. Results are expressed as percent MBP-positive area compared to control. Images were obtained using an Olympus BX-51 light microscope (Olympus, Richmond Hill, ON, Canada). The images were analyzed using Image-Pro Plus software (Version 6.1, Media Cybernetics, Inc., Silver Spring, MD, USA).

### 2.7. Statistical Analyses

All data were expressed as the mean ± the standard error of the mean (SEM). To compare experimental and control groups, a one-way ANOVA was performed followed by Dunnett's test. When statistically significant differences were found, Newman-Keuls test was used as post hoc test to determine the statistical difference between groups. All statistical analyses were performed with GraphPad Prism, Version 5 (GraphPad Software, Inc., San Diego, CA, USA). A *P* value of <0.05 was considered statistically significant. The figures were created using the GraphPad Prism 5 Statistical Analysis System.

## 3. Results

### 3.1. The Effect of Areca Nut Extract (ANE) on Spatial Working Memory

**Figure 1:**
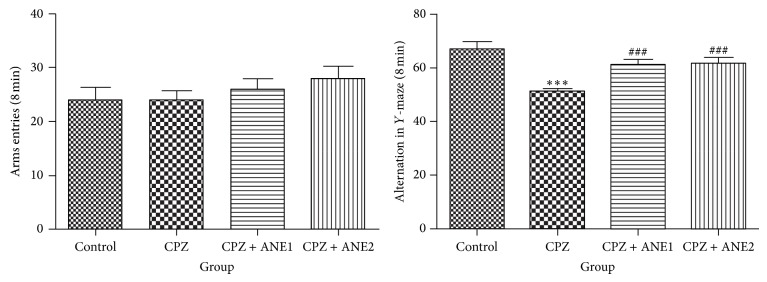
The effect of Areca nut extract (ANE) on spatial working memory in cuprizone- (CPZ-) induced mouse demyelination model. After 1 week of acclimation, male C57BL/6 mice were fed rodent chow containing 0.2% CPZ (w/w) for 8 weeks to induce demyelination. Mice in the ANE treatment groups were fed rodent chow containing 0.2% CPZ plus 1% ANE (ANE1) or 2% ANE (ANE2). The control mice received normal rodent chow. Data were expressed as the mean ± SEM. ^∗∗∗, ###^ *P* < 0.001. ^∗∗, ##^ *P* < 0.01. ^∗, #^ *P* < 0.05. ∗ indicates comparison with the control group while # indicates comparison with the CPZ group.

Spatial working memory was analyzed in mice subjected to demyelination by CPZ using the *Y*-maze test. The percentage of alternation behaviors significantly decreased in CPZ-treated mice compared with the control group (*P* < 0.001) (Figure 1). This finding indicated significantly impaired spatial working memory. Alternation in the *Y*-Maze significantly increased after treatment with both doses of ANE. These results suggest that ANE treatment improved spatial working memory.

### 3.2. The Effect of Areca Nut Extract (ANE) on Social Interaction Behaviors

**Figure 2:**
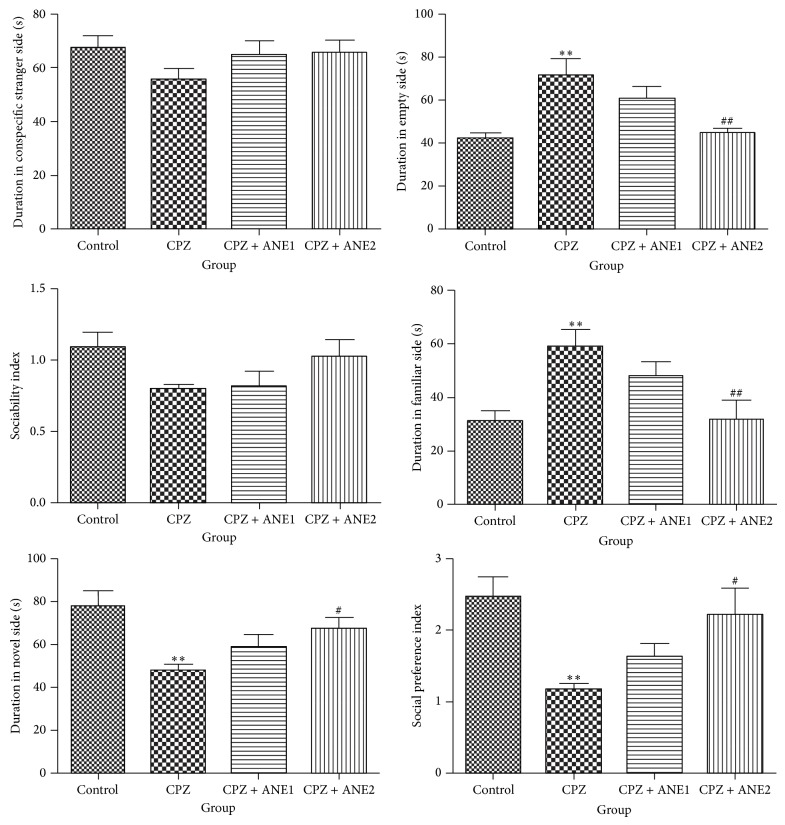
The effect of Areca nut extract (ANE) on sociability and social preference behavior tested in social interaction in cuprizone- (CPZ-) induced mouse demyelination model. After 1 week of acclimation, male C57BL/6 mice were fed rodent chow containing 0.2% CPZ (w/w) for 8 weeks to induce demyelination. Mice in the ANE treatment groups were fed rodent chow containing 0.2% CPZ plus 1% ANE (ANE1) or 2% ANE (ANE2). The control mice received normal rodent chow. Data were expressed as the mean ± SEM. ^∗∗, ##^ *P* < 0.01. ^∗, #^ *P* < 0.05. ∗ indicates comparison with the control group while # indicates comparison with the CPZ group.

It has been reported that animals subjected to CPZ-induced demyelination animals displayed less social interaction [13]. Stay durations on each side of a social test box were measured after a 5-min habituation period. In the sociability test, CPZ-treated mice spent less time in the compartment with a CSM compared with control group (Figure 2). ANE-treated mice showed a trend toward an increased duration with CSM. However, these changes were not statistically significant.

CPZ-treated mice spent more time (*P* < 0.01) in the EC compartment than control mice, and the mice treated with 2% ANE spend significantly less time in the EC compartment (*P* < 0.01), compared with CPZ-treated mice (Figure 2). The change in social interaction was expressed as a sociability index, defined as the ratio of duration of stay between CSM side and EC side, which showed ANE treatment inhibited the decrease in sociability induced by CPZ. However, these results were not statistically significant.

The social preference for novelty, which determines whether the test animal prefers socially novel versus familiar animals, was also investigated. CPZ exposure significantly increased the latency for engagement with socially familiar animals (*P* < 0.01). In addition, CPZ treatment significantly diminished the duration of time spent in the novel side of the apparatus compared with control mice (*P* < 0.01). These findings indicated significant lower social preference for CPZ-treated mice compared to control mice (*P* < 0.01). ANE treatment reversed the social preference impairment and increased the duration of time spent in the novel side and decreased the time spent on the familiar animal side. A statistically significant improvement was observed in the 2% ANE treatment group (*P* < 0.05) compared with the CPZ group (Figure 2). These results suggested that ANE treatment improved deficits in social interaction in mice subjected to CPZ-induced demyelination.

### 3.3. The Effect of Areca Nut Extract (ANE) on Matured Oligodendrocytes and Progenitor Cells

**Figure 3:**
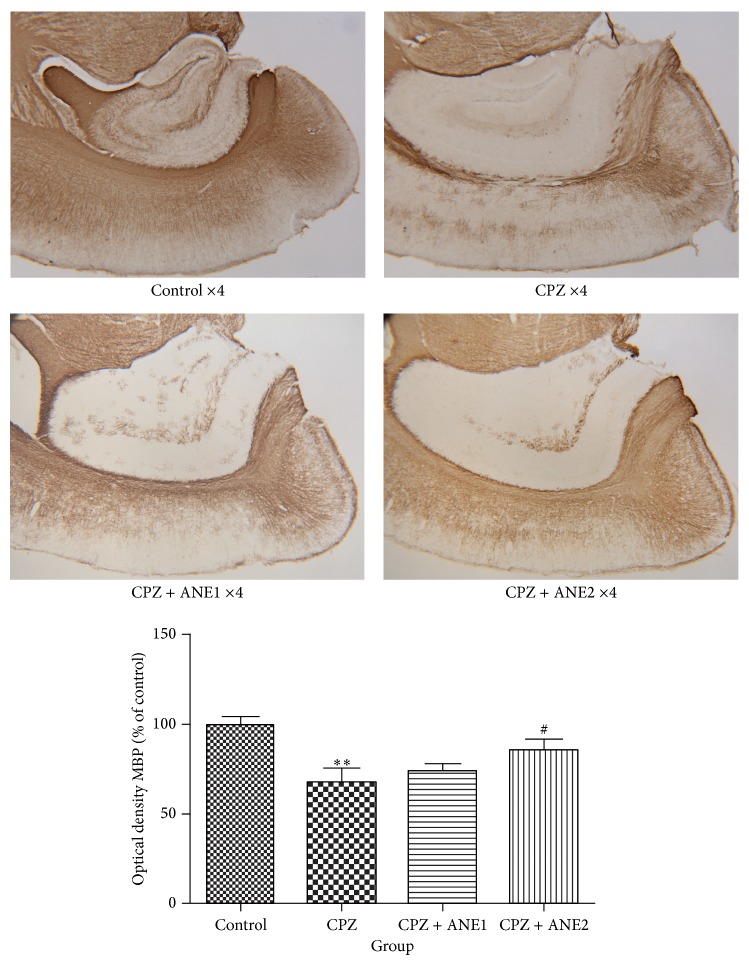
Areca nut extract (ANE) treatment may prevent the demyelination process in the frontal cortex. Photomicrographs showed MBP immunostaining in the frontal cortex in the control group, CPZ, CPZ + 1% ANE (ANE1), CPZ + 2% ANE (ANE2) groups, respectively. The graph shows the mean MBP-immunostaining optical density in the frontal cortex for each treatment group. Data are represented as the mean ± SEM. ^∗∗, ##^ *P* < 0.01. ^∗, #^ *P* < 0.05. ∗ indicates comparison with the control group while # indicates comparison with the CPZ group.

**Figure 4:**
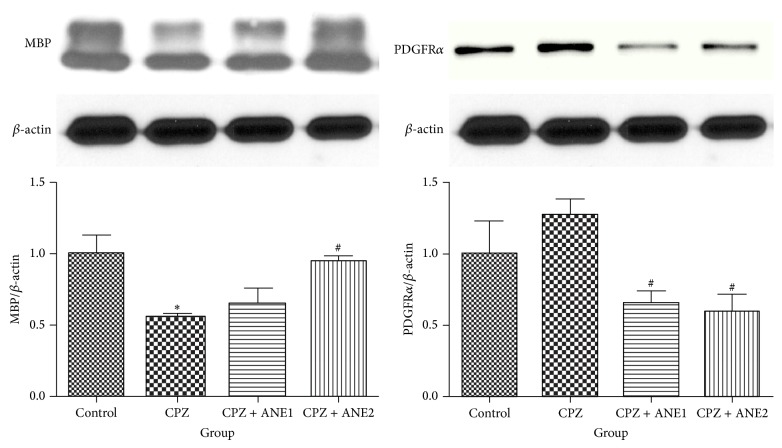
The effect of Areca nut extract (ANE) treatment on oligodendrocyte lineages. Figures show representative Western blot images of MBP and PDGFR*α*-immunoreactive bands from cortex samples of the treatment groups. Densitometric quantification shows the ratio of MBP and PDGFR*α* normalized to *β*-actin. Results shown are presented as the mean ± SD. ^∗∗, ##^ *P* < 0.01. ^∗, #^ *P* < 0.05. ∗ indicates comparison with the control group while # indicates comparison with the CPZ group.

MBP immunostaining results showed that CPZ-treated animals had significant myelin sheath disruption in the frontal cortex. The optical density of MBP was significantly decreased in the CPZ group compared to control animals (*P* < 0.01). The 2% concentration of ANE produced a statistically significant increase in MBP optical density compared to that of the CPZ-treated group (*P* < 0.05) (Figure 3). Western blot analysis showed that MBP protein expression in frontal cortex tissue decreased significantly (*P* < 0.05) after chronic CPZ exposure compared to control mice. ANE treatment tended to upregulate MBP expression, and compared to CPZ-treated mice, 2% ANE treatment showed a statistically significance increase in MBP expression (*P* < 0.05) (Figure 4). Treatment with 2% ANE produced obvious protective effects from CPZ-induced myelin sheath damage.

**Figure 5:**
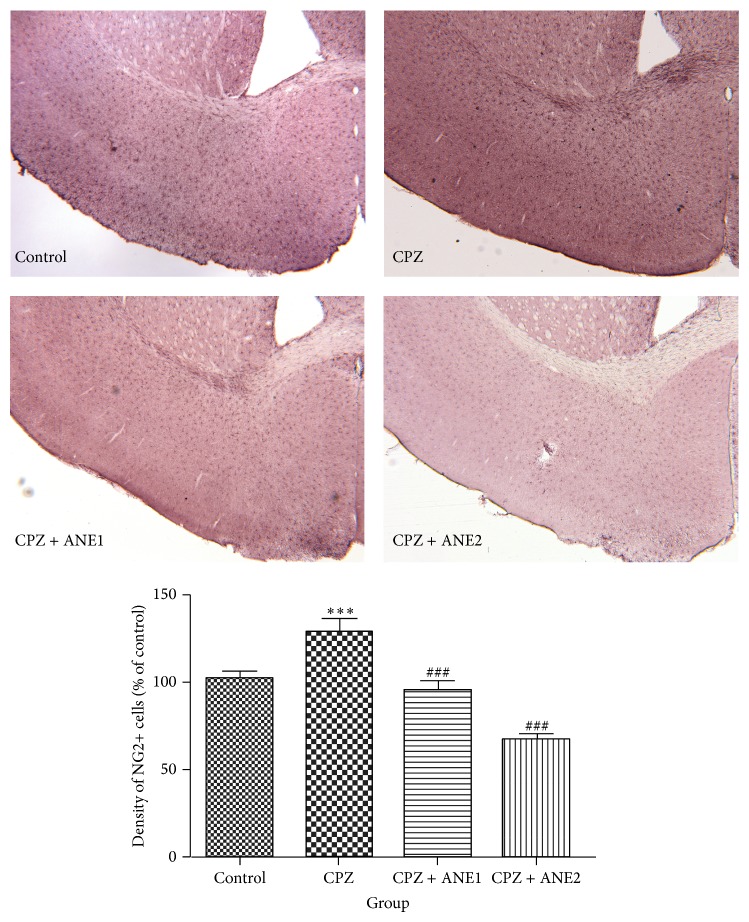
Areca nut extract (ANE) decreased NG2-positive cells in cortex. Photomicrographs show NG2-immunostaining in the frontal cortex of mice from the treatment groups. The bar graph shows the mean number of NG2-positive cells in the frontal cortex as a percentage of control. Data are represented as the mean ± SEM. ^∗∗∗, ###^ *P* < 0.001. ^∗∗, ##^ *P* < 0.01. ^∗, #^ *P* < 0.05. ∗ indicates comparison with the control group while # indicates comparison with the versus CPZ group.

**Figure 6:**
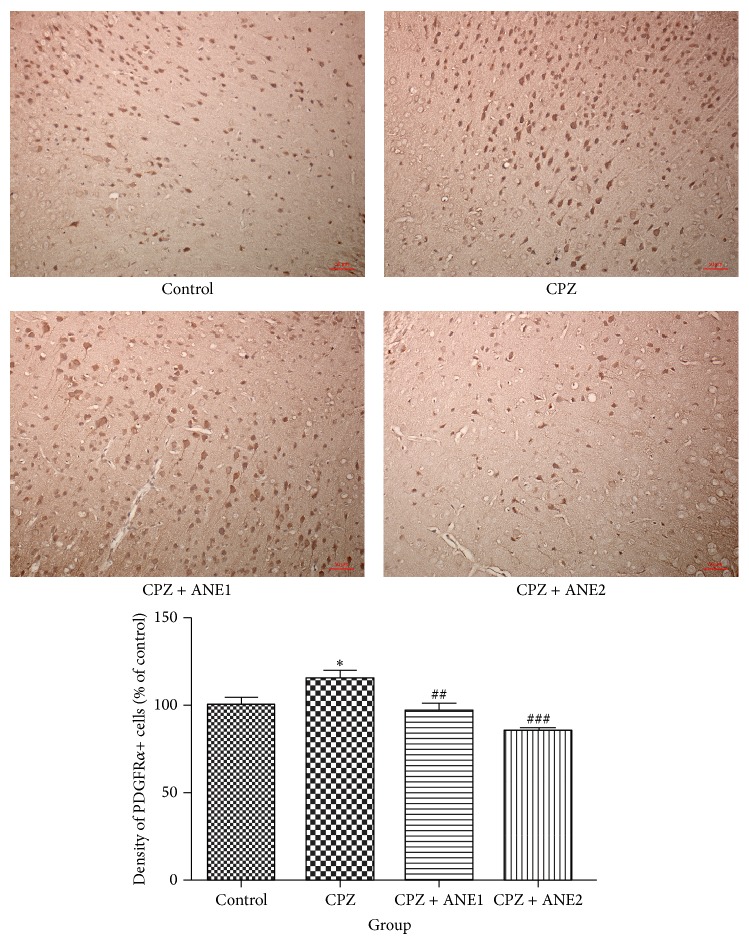
Areca nut extract (ANE) decreased PDGFR*α*-positive cells in the frontal cortex. The bar graph shows the mean number of PDGFR*α*-positive cells in the frontal cortex as a percentage of control. Data are presented as the mean ± SEM. ^∗∗∗, ###^ *P* < 0.001. ^∗∗, ##^ *P* < 0.01. ^∗, #^ *P* < 0.05. ∗ indicates comparison with the control group while # indicates comparison with the CPZ group.

NG2- and PDGFR*α*-positive cells are a group of OPCs that have the potential to develop into mature OLs in the adult brain [16]. After demyelination with CPZ, the numbers of NG2- and PDGFR*α*-positive cells in the cortex were dramatically higher (Figures 5 and 6) than those in the controls. The numbers of NG2- and PDGFR*α*-positive cells in the cortex of the ANE treatment group were significantly lower than in the CPZ group. Western blot analysis of cortex tissue also showed upregulation of PDGFR*α* protein expression, although the quantitative analysis did not show statistical significance. A significant decrease in PDGFR*α* protein expression was observed for both concentrations of ANE (*P* < 0.05, for both groups versus CPZ) (Figure 4). These results suggested that ANE might target OPCs and facilitate OPC differentiation into mature OLs to prevent myelin sheath damage.

### 3.4. The Effect of Areca Nut Extract (ANE) on AMP-Activated Protein Kinase (AMPK) Expression

**Figure 7:**
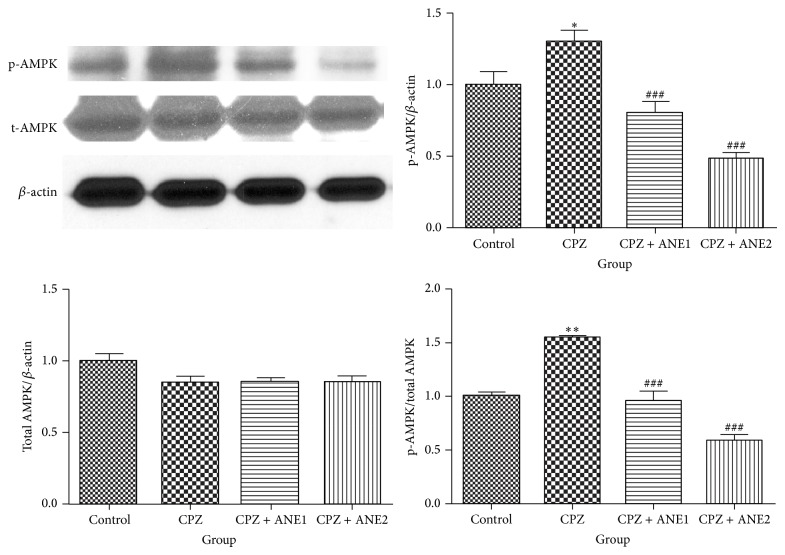
The effect of Areca nut extract (ANE) treatment on AMP-activated protein kinase (AMPK) expression. Representative immunoblots of AMPK expression in cortex samples are shown. Densitometric quantification shows the ratio each protein expression normalized to *β*-actin expression. Results are presented as the mean ± SD. ^∗∗∗, ###^ *P* < 0.001. ^∗∗, ##^ *P* < 0.01. ^∗, #^ *P* < 0.05. ∗ indicates comparison with the control group while # indicates comparison with the CPZ group.

It has been demonstrated that Areca nut constituents modulate metabolic signals regulating a crucial stress-sensing enzyme, AMPK [18]. Protein expression in cortical specimens was analyzed to determine the effect of ANE on AMPK kinase. Phosphorylated AMPK*α* was significantly increased in mice subjected to CPZ-induced demyelination compared with control mice (*P* < 0.05). After treatment with 1% and 2% ANE, phosphorylated AMPK*α* ratios were significantly reduced compared with the CPZ group (*P* < 0.001; *P* < 0.001, resp.). Total AMPK expression did not show significant differences between the groups (Figure 7). The data suggested that ANE treatment might suppress AMPK activation.

## 4. Discussion

The focus of this study was the neuropathological features of white matter in schizophrenic brains and animal models designed to mimic this neuropathology. With low-dose CPZ administration, rodents showed selective demyelination lesions in the prefrontal cortex, which made this model especially attractive for schizophrenia studies [17, 19, 20]. Previous studies have shown CPZ-induced demyelination in mice resulted in deficits spatial working memory, social interaction, and executive function. The abnormal behaviors, demonstrable demyelination, and oligodendrocyte loss observed in CPZ-treated mice suggested a correlation between white matter damage and certain behavioral abnormalities [13, 21–23].

Impairment of working memory has been widely reported in patients with schizophrenia [24]. Some studies have indicated that impaired spatial working memory has a strong correlation with negative symptoms of schizophrenia and could be considered an endophenotype of schizophrenia [21]. In the *Y*-maze test, ANE significantly enhanced spatial working memory in CPZ-treated mice. This was consistent with the findings of Joshi et al. [25], which suggested that* Areca catechu* extract produced a greater increase in spatial memory and learning owing to a higher content of arecoline. In addition, the phenolic fraction such as hydroxychavicol was also found to improve cognitive deficits [26].

A previous study showed that CPZ-treated mice demonstrated deficits in social interaction starting at 4 weeks after CPZ exposure when demonstrable loss of myelin sheath and MBP were observed [13]. These findings suggested that normal behavior in the social interaction test relied on intact white matter. Our results showed less social ability and social preference in mice treated with CPZ for 8 weeks. ANE treatment improved social interaction time in CPZ-treated mice. MBP immunostaining showed obvious demyelination in the cortex after 8 weeks of CPZ treatment. Western blot analysis also demonstrated decreased expression of MBP. ANE showed protective effects on myelin degradation and increased MBP expression in the cortex.

During dosing with CPZ, mature OLs undergo apoptosis. OPCs proliferate during recovery in the subventricular zone, migrating to the demyelinated areas of the CC and cortex, and develop into mature OLs [21]. In animals subjected to demyelination, a dramatic increase in NG2-positive cells was observed in the frontal cortex and CC, but not in the subventricular zone. These results indicate that the migration of NG2-positive cells to the demyelinated areas of the cortex and CC occurred at a late stage of demyelination [16]. During the recovery period, the number of NG2-positive cells was reduced with increasing recovery time [21]. PDGFR*α* and NG2 antibodies were used to detect OPCs. In our study, PDGFR*α*-positive and NG2-positive OPCs increased in the cortex of mice subjected to CPZ-induced demyelination. ANE treatment reduced the numbers of PDGFR*α*-positive and NG2-positive OPCs in the frontal cortex. It has been reported that the Areca nut possesses antioxidative effects [5]. This antioxidative capability of ANE might help to prevent myelin damage, subsequently decreasing the compensatory increase of NG2 and PDGFR-*α*. Myelin-forming cells in the central nervous system can arise from OPCs under certain conditions. Exposure of OPCs to PDGF led to continuous self-renewal without differentiation. In contrast, OPCs not exposed to mitogens differentiate into oligodendrocytes and do not proliferate [27]. The Western blot results indicated that ANE decreased platelet-derived growth factor receptor (PDGFR*α*) expression and thus might arrest OPCs proliferation and stimulate differentiation into oligodendrocytes. Although a search of the literature did not produce any publications describing a direct effect of ANE on NG2 and PDGFR-*α*, a future study investigating the possible effect of ANE on NG2 and PDGFR-*α* in the mice without a cuprizone-diet is necessary to evaluate the possible direct effect of ANE on NG2 and PDGFR-*α*. Moreover, further studies are necessary to elucidate the effects of ANE on spontaneous remyelination after cuprizone-diet at different time points.

AMPK is a serine/threonine protein kinase. Activation of AMPK through oxidation in the cerebellum may contribute to neurodegeneration, and AMPK signaling pathways have been proposed as therapeutic targets for the treatment of neurodegenerative diseases [28]. In our study, chronic administration of CPZ caused activation of AMPK shown as increased phosphorylation level of AMPK*α* (p-AMPK*α*), and different concentration of ANE suppressed AMPK activation. A greater role for AMPK in regulating cell growth and proliferation has been proposed based on recent studies [29]. AMPK activating agents cause dephosphorylation of glycogen synthase kinase-3*β* (GSK-3*β*) [30]. GSK3*β* was found to suppress oligodendrocyte differentiation and myelination in vivo [31]. Alterations in GSK-3*β* and *β*-catenin have been reported in schizophrenic patients and have been targeted by antipsychotic drugs [32, 33]. In vitro studies have demonstrated that ANE treatment induced the phosphorylation of GSK-3*β* [34–36]. *β*-catenin expression was increased by arecoline in a dose-dependent manner [37]. The results of this study suggest that ANE treatment may facilitate OPC differentiation and myelination processes and that the AMPK signaling pathway may be involved in this process.

## 5. Conclusion

This study showed that ANE treatment may enhance cognitive ability and social activity by facilitating OPC differentiation and protecting myelin damage in cortex. The AMPK signaling pathway may be involved in this process. Given the limitations of the current study, detailed pharmacological investigations may be required to (1) identify the profile of ANE by using HPLC and determine the active components responsible for the preventative effect on white matter demyelination and (2) reveal whether arecoline and phenolic compounds in ANE attenuate demyelination and enhance oligodendrocyte development synergistically and whether they have dual effects: a biphasic dose-response relationship.
